# Use of Permanganate of Potassa in Chronic Otorrhœa*The greater part of this paper was presented at the last meeting of the American Otological Society.

**Published:** 1879-08

**Authors:** Lucien Howe


### ON THE USE OF PERMANGANATE OF POTASSA IN CHRONIC OTORRHŒA.*

BY LUCIEN HOWE, M. D.

So many different substances have been recommended for chronic otitis media purulenta, that it would seem unnecessary to add to the number. The one to which I would call attention, however, is so frequently employed for similar purposes in other portions of the body, that there would seem to be nothing new in its application to the ear. It has been incidentally referred to in this connection by one or two, but I am not aware that Von Troltsch, Gruber, Roosa, or any other writers, consider it worthy of mention. It would be strange, however, if among the numerous astringents or caustics used for this purpose, so reliable an agent as the permanganate of potassa, had not already been thoroughly and systematically tried.

* The greater part of this paper was presented at the last meeting of the American Otological Society.

I simply propose, therefore, to cite a few cases in which it was used, and compare the result with that obtained in the employment of other agents.

For this purpose I have collected from my case book the records of 53 individuals thus treated. Several others were noted, which would have been included in the list, but for the fact that the records were not complete, or that the patients were lost sight of before any definite conclusions could be arrived at.

The ages of the patients ranged from two to forty-four years, and the disease had lasted, with greater or less variation, from three weeks to twenty-two years. In several instances where the otorrhœa followed a primary acute otitis media purulenta, it was not easy to say when the discharge had passed into a condition to deserve the term "chronic." I have, therefore, excluded all cases in which the disease has not lasted at least three weeks, or which have still presented those symptoms of pain, etc., which would entitle them to the term "acute."

Concerning the etiology of these cases, there was about the same variety that one would expect in a series chosen at random. Scarlatina furnished its usually large quota of almost half—more exactly twenty-one. Seven followed primary acute otorrhœa. Two resulted from measles, and a number were dependent upon causes which it was impossible to ascertain.

The principal treatment prescribed in all of these, was the use of a solution of permanganate of potassa in water. In the milder cases two grains to the ounce was found to be sufficient, but if more severe, or of very long duration, the strength was increased to four, six, or even eight grains to the ounce.

The patient was simply instructed to syringe out any accumulated secretion with tepid water, and then pour a few drops of the fluid into the ear. This was allowed to remain five or ten minutes if it produced no smarting or burning sensation. If decided inconvenience followed, however, it was washed out sooner. When the discharge was at all abundant, this was repeated twice daily, and if very profuse, of course the ear was kept clean by more frequent washings with water alone. Whenever polypi developed, as was the case in three instances, or any other complications presented, they were treated by the usual methods.

A sufficient time has now elapsed to count with considerable certainty on the effects produced; and as a result, I find:—of the 53 cases 40 have entirely recovered in an average time of 38 days. In six the discharge recurs at intervals; in four it is continual, but lessened, as to quantity and the fetid character of the odor; in three, it still persists.

Now comparing this treatment with those methods usually placed foremost in the text books, I find the results are decidedly in its favor. In seven of these cases, nitrate of silver had been used after the manner recommended by Schwartz, and although an improvement was manifest in most of them, the progress was not as rapid as under the use of permanganate of potassa. Moreover, in ten other cases treated with nitrate of silver the discharge was arrested in six, after an average of 51 days; two persisted in the treatment about six months and then disappeared; while concerning two, the record is imperfect. In three of the cases reported, I began by using sulphate of copper, but after an average of six days changed to the permanganate of potassa, and improvement followed quite as rapidly, to say the least, as before. Again, in five other cases treated with sulphate of copper alone, I find, on the average, the discharge continued for 63 days. Sulphate of zinc, alum and tannic acid, alcohol, carbolic acid and other remedies have also been used in occasional instances, but the record concerning them is not sufficiently definite to warrant any exact statement. There are obvious objections against drawing any very positive conclusions from the data here given, to which it is only fair to direct attention. The principal one is, that the number of cases reported is quite limited. Again, it is probable that many cases of otitis media purulenta will yield to the use of almost any astringent, or of water alone, if persistent effort is continued. In spite of these objections, however, the facts just cited indicate that in the permanganate of potassa we have a remedy quite as efficacious, if not more so, than those upon which reliance is usually placed. It has in addition certain advantages which would appear to make it preferable to most other applications. Unlike the strong solutions of nitrate of silver, it can easily be used by the patient, and is not followed by as much smarting or pain. Especially is it to be preferred for the antiseptic or deodorizing quality by which the fetid smell of the secretion is removed to an extent not observed in the use of any other application, not excepting carbolic or salicylic acids. In general, therefore, it seems to be a remedy for this disease, which can be counted on to heal safely, quickly and easily.

